# Guiding AI in radiology: ESR’s recommendations for effective implementation of the European AI Act

**DOI:** 10.1186/s13244-025-01905-x

**Published:** 2025-02-13

**Authors:** Elmar Kotter, Tugba Akinci D’Antonoli, Renato Cuocolo, Monika Hierath, Merel Huisman, Michail E. Klontzas, Luis Martí-Bonmatí, Matthias Stefan May, Emanuele Neri, Konstantin Nikolaou, Daniel Pinto dos Santos, Maija Radzina, Susan Cheng Shelmerdine, Arianna Bellemo, European Society of Radiology (ESR)

**Affiliations:** 1 Department of Diagnostic and Interventional Radiology, Medical Center-University of Freiburg, Freiburg im Breisgau, Germany; 2 Faculty of Medicine, University of Freiburg, Freiburg, Germany; 3 Cantonal Hospital Baselland, Liestal, Switzerland; 4 Department of Medicine, Surgery, and Dentistry, University of Salerno, Fisciano, Italy; 5 European Society of Radiology (ESR), Vienna, Austria; 6 Department of Radiology and Nuclear Medicine, Radboud University Medical Center, Nijmegen, The Netherlands; 7 Department of Radiology, School of Medicine, University of Crete, Heraklion, Crete, Greece; 8 Medical Imaging Department and Biomedical Imaging Research Group at Hospital Universitario y Politécnico La Fe and Health Research Institute, Valencia, Spain; 9 Department of Radiology, University Hospital Erlangen, Friedrich-Alexander-Universität Erlangen-Nürnberg, Erlangen, Germany; 10 Department of Translational Research, University of Pisa, Pisa, Italy; 11 Department of Diagnostic and Interventional Radiology, Eberhard Karls-University Tuebingen, Tuebingen, Germany; 12 University Hospital of Cologne, Cologne, Germany; 13 University Hospital of Frankfurt, Frankfurt, Germany; 14 Radiology Department, Riga Stradins University, Riga, Latvia; 15 University of Latvia, Medical and Natural Sciences Faculty, Riga, Latvia; 16 Department of Clinical Radiology, Great Ormond Street Hospital for Children, London, UK; 17 UCL Great Ormond Street Institute of Child Health, London, UK; 18 NIHR Great Ormond Street Hospital Biomedical Research Centre, London, UK; 19 Am Gestade 1, Vienna, Austria

**Keywords:** Artificial intelligence, Regulation, Implementation, AI literacy, Human oversight

## Abstract

**Abstract:**

This statement has been produced within the European Society of Radiology AI Working Group and identifies the key policies of the EU AI Act as they pertain to medical imaging. It offers specific recommendations to policymakers and the professional community for the effective implementation of the legislation, addressing potential gaps and uncertainties. Key areas include AI literacy, classification rules for high-risk AI systems, data governance, transparency, human oversight, quality management, deployer obligations, regulatory sandboxes, post-market monitoring, information sharing, and market surveillance. By proposing actionable solutions, the statement highlights ESR’s readiness in supporting appropriate application of the AI Act in the field, promoting clarity and the effective integration of AI technologies to ensure their impactful and safe use for the benefit of Europe’s patients.

**Critical relevance statement:**

With the impending arrival of the EU AI Act, it is critical for stakeholders to provide timely input on its key areas. This statement offers expert feedback on the aspects of the EU AI Act that will affect medical imaging.

**Key Points:**

- The AI Act will significantly impact the field of medical imaging, shaping how AI technologies are used and regulated.
- The ESR is committed to develop guidelines and best practices, collaborating on the implementation process.
- This statement offers expert feedback on the aspects of the framework that will affect medical imaging.

**Graphical Abstract:**

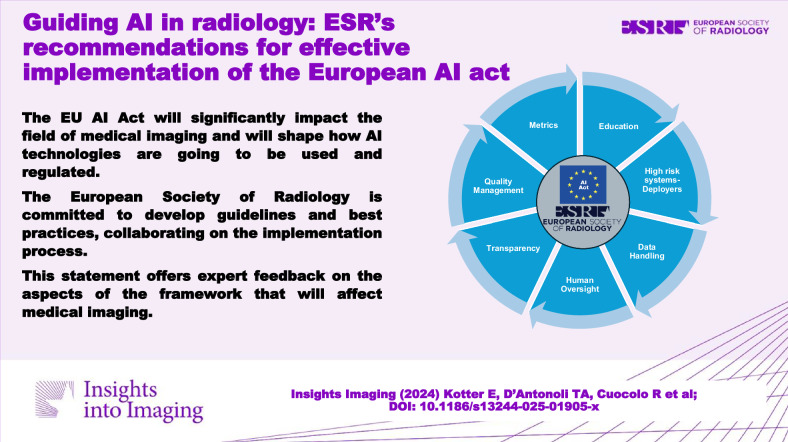

## Introduction

**Fig. 1:**
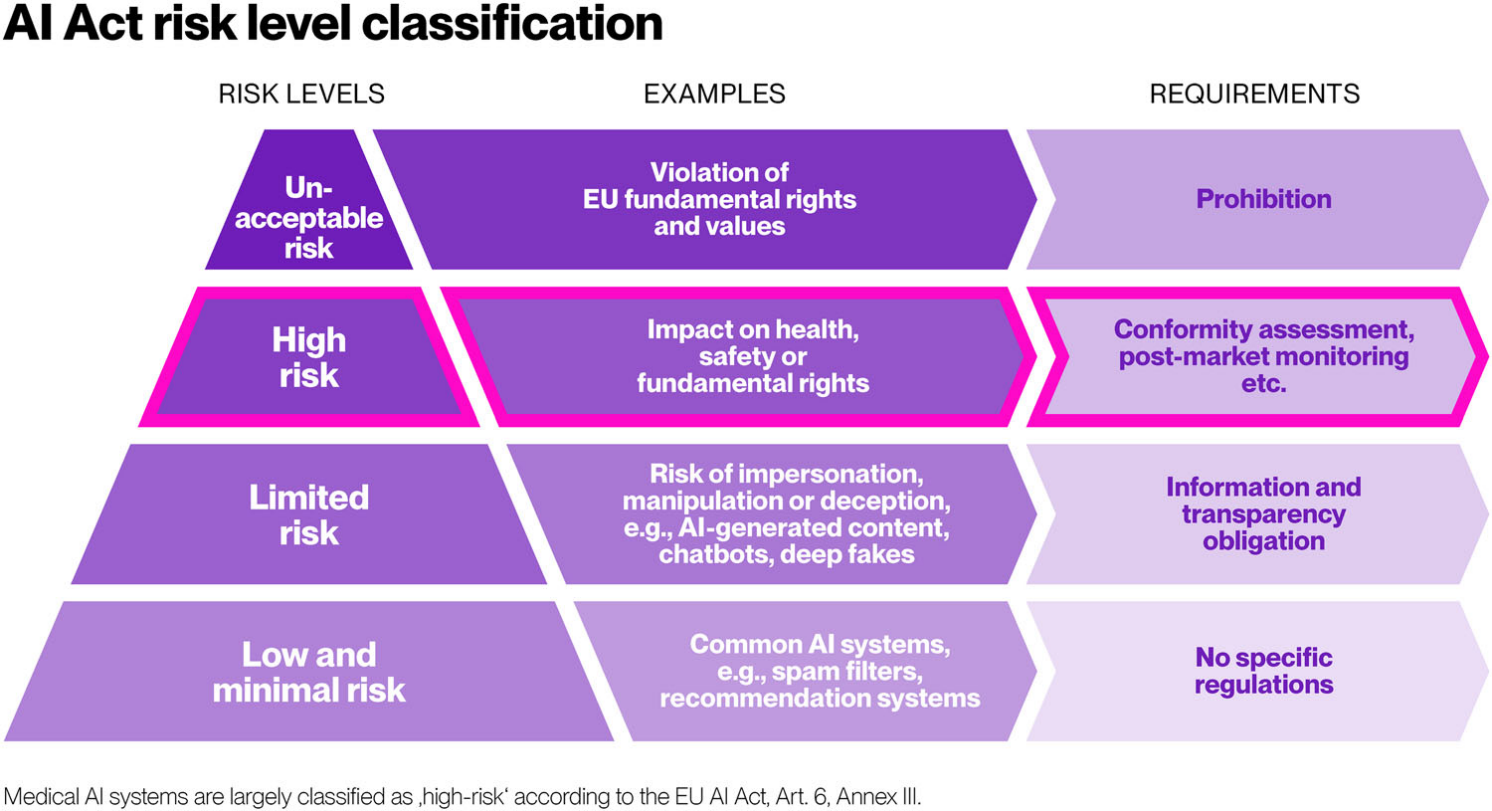
AI Act risk level classification

The European Society of Radiology (ESR), as the leading European voice in the field of medical imaging, is actively engaged in the ongoing dialogue surrounding the Artificial Intelligence Act (AI Act) [1] (Fig. 1). The ESR has also focused on this legislation in the past, both through statements from the organisation and as part of multi-stakeholder efforts, underscoring its commitment to this regulatory framework [2, 3]. Recognising the significant potential this legislation holds for medical imaging, as ESR’s AI Working Group, we are eager to contribute our insights and recommendations and to be considered a key stakeholder in developing implementation guidance by the European Commission.

AI innovation and integration have become a central priority for research and development in the field of radiology, driven by the significant advancements and applications of AI in this area of healthcare [4]. At the time of writing over 200 CE-certified AI applications targeting medical imaging are available [5, 6]. The utilisation of AI technologies in our specialty is poised to revolutionise diagnostic processes [7], enriching image interpretation and ultimately elevating standards of patient care. Significant impacts are especially anticipated in the areas of cardiovascular diseases and oncology, where imaging plays a central role. Given this transformative potential, the ESR provides the following recommendations to facilitate the clinical implementation of the AI Act by European institutions.

The AI Act represents a pivotal opportunity to establish clear guidelines for the seamless integration of AI into medical imaging practices. By prioritising safety and reliability, we are committed to ensuring thorough evaluation and validation of AI technologies before and after their clinical deployment. Standardised protocols for development and implementation will maximise the benefits of AI for patients, the healthcare system, and society at large.

The following presents the ESR’s analysis of the key policies of the AI Act identified as relevant within the context of radiological practice and beyond. Produced by a group of radiologists with expertise in AI, it aims to provide valuable insights that will guide the development of effective implementation strategies. Each section of this document reflects a collaborative effort, with specific areas refined within smaller working groups. Consensus on the final recommendations was reached through plenary meetings involving all group members. In conclusion, the group has identified potential gaps and uncertainties in the legislation and proposes solutions to address these (summarised in Table 1), ensuring that the AI Act facilitates the responsible and beneficial integration of AI in radiology while upholding the highest standards of patient care and safety.

**Table 1:** Overview of recommendations

Theme	Article	Recommendation and related action	Responsible Actor/s
Education	Article 4 Article 3 (46)	Support the development of training programs to empower both radiology personnel and patients with the knowledge and skills necessary for safe and effective engagement with AI technologies in radiological practice. Expand radiologists’ training to cover comprehensive AI education.* Call for the implementation of regular training for radiologists to prevent deskilling and ensure continuous competency.*	ESR’s relevant subcommittees, patient outreach and charity groups.
High-risk AI classification and clinical decision support	Article 6, Annex III	There are diverse applications of AI in medical imaging. ESR is committed to collaborating with the European Commission to develop guidelines and practical examples specific to AI use in radiology.	ESR in collaboration with the European Commission.
Data handling	Article 10	Support the formulation of guidelines and optimal strategies for data handling in AI applications.	ESR in collaboration with European Societies of Radiology subspecialties and national societies.
Human oversight	Article 14	Invest in research on mental load in healthcare to set effective human oversight thresholds.	European research funding Bodies, Universities, and psychology organisations.
		*See Education recommendations and related actions.	
Transparency	Article 13	Support the elaboration of correct terms, application rules, and standards for AI systems in medical imaging	ESR in collaboration with European Societies of Radiology subspecialties, national societies, and the European Commission.
		Recommend using AI model cards to increase transparency, providing detailed information on a model’s development, capabilities, and limitations.	
Quality management system (QMS)	Article 17	Harmonise Quality Management System requirements between the European Union Medical Device Regulation and the AI Act, allowing for unified documentation for ‘high-risk’ AI tools, streamlining compliance and ensuring clarity in medical imaging implementation.	European Commission, Regulatory Bodies, and Notified Bodies.
Deployers and high-risk AI systems	Article 26	Support deployers with recommendations and monitoring strategies of high-risk AI systems.	ESR in collaboration with providers and deployers.
		Collaborate in the specification of the obligations of deployers in medical imaging.	
Clinical studies	Article 57	Call for the requirement of large-scale, high-quality clinical studies governed by EU regulations for any AI product affecting patient health, and oversight by competent authorities with AI expertise.	Funding bodies and European Commission.
Metrics and post-market surveillance	Article 72 Article 34	Support the collection of performance metrics over larger populations, alleviating the burden on end-users.	ESR in collaboration with providers and deployers, national societies, notified bodies and the European Commission.
		Assist the facilitation of post-market surveillance through interoperable and technical solutions.	
		Establish infrastructures to track AI usage and potential adverse effects.	

## Definitions

To establish a clear understanding of key terms relevant to the AI Act, several definitions outlined in Article 3 of the regulation are foundational.

- An ‘AI system’ is described as a machine-based system designed to operate with varying levels of autonomy, which may demonstrate adaptiveness after deployment. These systems infer, based on the input they receive, how to produce outputs—such as predictions, content, recommendations, or decisions—that can impact physical or virtual environments, either for explicit or implicit objectives.
- The term ‘provider’ refers to any natural or legal person, public authority, agency, or other entity that develops an AI system or general-purpose AI model, or has one developed, and subsequently places it on the market or puts it into service under its name or trademark, whether as a paid or free offering.
- Meanwhile, a ‘deployer’ is any entity, except individuals using AI for personal, non-professional purposes, that uses an AI system under its authority. In the radiological context, a deployer may be a hospital, clinic, or radiology practice that integrates and uses AI systems in its workflow.
- Finally, the term ‘operator’ encompasses providers, product manufacturers, deployers, authorised representatives, importers, or distributors, highlighting the interconnected roles within the AI lifecycle. These definitions frame the legal responsibilities and relationships within the AI ecosystem.

## Methods

The ESR AI Working Group was formed by the European Society of Radiology (ESR) to provide expert input on the EU AI Act. Composed of radiologists with expertise in AI, the group worked collaboratively on drafting this statement. For the purposes of this statement, “we” refers to the ESR throughout the paper, as we were tasked by ESR to undertake this work on behalf of ESR. During the period from April to September 2024, regular virtual meetings were held every two to three weeks. Agendas, presentations, and drafts were stored in a dedicated shared online space, accessible to all members to facilitate collaboration.

An initial brainstorming session identified nine key articles of the AI Act that were particularly relevant to medical imaging, such as post-market surveillance, data governance, and risk classification. Members selected specific policy areas based on their expertise and were later organised into smaller focus groups of two to three individuals to draft the sections.

Iterations of the drafts were reviewed during plenary meetings, incorporating feedback from all members. Recommendations were refined and finalised through plenary discussions, achieving consensus. The final draft was reviewed and approved by the ESR Board of Directors.

## AI literacy

The AI Act Article 4 underscores the critical importance of AI literacy for all stakeholders, including healthcare providers, in the development, deployment, and use of AI systems. The ESR recognises the transformative potential of AI in enhancing diagnostic accuracy and efficiency. However, it is equally important to ensure that both our workforce and our patients are adequately informed about AI technologies.

As defined by the AI Act, in Article 3 (56) ‘AI literacy’ means “skills, knowledge and understanding that allows providers, deployers and affected persons, taking into account their respective rights and obligations in the context of this Regulation, to make an informed deployment of AI systems, as well as to gain awareness about the opportunities and risks of AI and possible harm it can cause.” This applies not only to providers and deployers but also to patients, as “affected persons” whose medical management may be influenced by AI outputs. Integrating AI literacy into patient care involves informing patients about the use of AI and how AI impacts their imaging exams. One critical question is whether this should be part of the existing informed consent process for imaging exams or a separate consent specifically for AI use. This issue is further compounded by the wide range of potential applications of AI in medical imaging, some of which may be integral to the medical procedure. In this setting, communicating clearly with the patient is essential for maintaining trust and transparency in patient care. We acknowledge that the approach to consent for AI use depends heavily on varying domestic medical-legal requirements, and while we advocate for a simplified process to avoid complexity to the detriment of patients’ understanding, the issue extends beyond our direct influence and remains a priority for future consideration and dialogue.

The ESR aims to promote AI literacy and enhance public awareness of AI benefits, risks, safeguards, rights, and obligations. The ESR plans to take action in developing a code of conduct for AI in imaging for the radiology workforce in cooperation with relevant stakeholders, including national radiology societies, to guarantee a harmonised approach across Europe. The ESR is also prepared to support the development of codes of conduct for other medical specialties.

Furthermore, the ESR advocates for the increased integration of AI literacy into medical school and residency training curricula. We support the inclusion of AI training as a critical component of radiology residency training, as this can be seen in our recommended training curricula [8]. This will equip future doctors with the knowledge and skills needed to utilise AI effectively and ethically. We also believe that the existing workforce should be further qualified through continuous medical training, and the ESR is committed to playing a key role in upskilling the current radiology workforce [9, 10]. Teaching radiology using AI tools is also essential, as it enables students and professionals to acquire advanced and precise skills in a faster and more interactive way.

### Recommendation

The AI Act’s focus on AI literacy aligns with ESR’s commitment to advancing the safe and effective use of AI in radiology. The ESR offers to take a leading role in any efforts to establish references for levels of AI literacy required for the workforce to safely deploy and use AI in medical imaging. We pledge to support ongoing training efforts for radiology personnel as well as educational campaigns for patients, ensuring that everyone involved is well-informed and prepared to engage with AI systems and implementation of these systems in radiological practices. The ESR patient advisory group will engage patients in the development process for AI training and education.

## Classification rules for high-risk AI systems

According to the EU AI Act, products which are regulated by the European Union Medical Device Regulation (EU MDR), automatically fall under the “high-risk AI” category defined in Article 6 and Annex III. Medical devices, which may affect patient outcome and quality of life, are inherently high-risk and require oversight by radiologists or similarly trained domain experts in imaging. However, Section 3 of Article 6 states that specific use cases may be exempt from this requirement. These exemptions include tasks such as performing a narrow procedural task, preparatory tasks for human checking, improving activities previously undertaken by humans, or alerting to deviations from prior decisions.

In radiology, such tasks could involve organising unstructured data or improving the language, consistency, and tone of text in reports. While these applications may seem harmless and a good application for so-called general-purpose AI, it is well known that these models are still prone to errors (also known as hallucinations), even when the output is plausible to a human user. These errors can change the nuance of a report, potentially affecting downstream clinical decision-making. Evaluating this effect is challenging, as decision-making processes are rarely monofactorial and may include human-machine interactions that need further investigation and development.

Nevertheless, other tools for workflow optimisation (e.g., automated viewing protocols) might not be classified as high-risk due to their narrow, procedural nature, allowing for human oversight before significant clinical decisions are made. Access to these tools could greatly benefit radiology practices amid rising demand and limited resources. Thus, it is crucial to have a clear understanding of which use cases are free from legal ambiguity under the AI Act, given the resource-intensive nature of compliance. In this setting, special consideration should be given to radiology triage tools that prioritise imaging cases based on urgency for human interpretation. These should rightly be classified as high-risk because they can influence decision-making and potentially cause harm. Their impact on fundamental human rights, particularly the right to freedom from discrimination and the right to equality, should also be considered. Even if the risk of harm and decision-making can be minimised, certain patients or groups may be prioritised over others, leading to unequal treatment. Clarification is needed as to whether imaging triage tools are comparable to emergency healthcare patient triage systems mentioned in Annex III and if they warrant additional scrutiny compared to other high-risk AI applications. Specifically, this application and designated applications for workflow optimisation should be included in the “comprehensive list of practical examples of use cases of AI systems that are high-risk and not high-risk” mentioned in paragraph 5 of Article 6.

### Recommendation

Considering the range of possible applications for AI in medical imaging, the ESR recommends and plans to develop guidelines and practical examples pertaining to the use of high-risk AI applications in radiology for consideration by the European Commission in its implementation guidance. Early involvement of stakeholders is paramount to ensure the safe deployment of AI medical devices into clinical practice in accordance with the AI Act, whether high-risk or not. In particular, the ESR encourages consultation of domain experts in the creation of “guidelines specifying the practical implementation” of Article 6 and related practical examples for high-risk and non-high-risk AI, which are to be made available within 18 months from the date of entry into force of the AI Act, as mentioned in Article 6, paragraph 5. In this context, guidelines for the correct framing of triaging tools in medical imaging, including the emergency setting, are specifically required to clarify the framing of such AI medical devices to the radiology community represented by the ESR.

## Data and data governance

The ESR appreciates the focus on data governance and data quality outlined in Article 10 of the AI Act. This emphasis is of paramount importance to ensure the accuracy, fairness, and safety of AI applications in medical imaging, aligning with our commitment to high standards in data management and ethical AI deployment [11].

The AI Act stipulates that high-risk AI systems must be developed using datasets that meet strict quality criteria. These criteria, detailed in paragraphs 2 to 5 of Article 10, include comprehensive data governance and management practices. In radiology, this means ensuring that AI systems are trained on datasets that are as relevant and representative as possible. These datasets must reflect the geographical or contextual settings where the AI system will be used, enhancing the system’s reliability and applicability in diverse clinical environments. These requirements can only be fulfilled with the availability of corresponding high-quality data. The European Health Data Space (EHDS) will enable the constitution of general data protection regulation (GDPR)-compliant datasets, which will require prompt implementation. Therefore, ESR considers the establishment of the EHDS of utmost importance for the future development of medical AI systems and is willing to contribute to its implementation. Of key importance is the EUropean Federation for CAncer IMages (EUCAIM) [12], the cornerstone of the European Cancer Imaging Initiative, establishing a comprehensive pan-European image-centric digital federated infrastructure with millions of medical images to foster AI systems development and validation. The AI Act also addresses the processing of special categories of personal data to detect and correct biases in high-risk AI systems. This is subject to strict safeguards to protect fundamental rights and freedoms. In radiology, this means that if bias detection and correction cannot be achieved with other data types, special categories of personal data may be used under rigorous conditions, including de-facto anonymisation, strict data access controls, and data deletion when no longer necessary.

### Recommendation

In line with these provisions, robust data governance frameworks need to be developed and implemented within the medical imaging community. The ESR is willing to take a proactive role in developing guidelines and best practices for data management in AI applications, while also committing to supporting the EHDS implementation for future development of AI systems. This initiative will be coordinated with national radiology societies to ensure a cohesive and comprehensive approach across Europe.

## Transparency and provision of information to deployers

Article 13 of the AI Act mandates that high-risk AI systems must be designed and developed with sufficient transparency to ensure that deployers can accurately interpret the system’s output and use it correctly. To achieve this, providers are required to supply detailed instructions for safe use—such as the system’s intended purpose, performance characteristics, known limitations, human oversight measures, maintenance required and technical capabilities—that are concise, complete, correct, clear and accessible to deployers. To enhance relevancy, accessibility, trustworthiness, and comprehensibility, providers can outline key information and instructions in the form of so-called model cards, which include the relevant demographics (age, gender, ethnicity, comorbidities, etc.) of the data used to train a model. Details on the expected accuracy and robustness of the system must be provided, including expected risks of misuse or misinterpretation and the intended purpose of the system. Deployers must be clearly informed about the intended user of the AI system (e.g., doctor, nurse, technician, etc.), and provided with insights on the type of data required for its proper use. The provider must ensure the interpretability of system outputs and results according to the state of the art (see also Article 14, Human oversight measures). Methods and standards for transparency and explainability remain an area of ongoing development and research.

### Recommendation

The ESR offers to take part in any efforts to further elaborate on the correct terms and rules of application of AI systems in medical imaging as well as setting standards for the expected performance and limitations of high-risk AI systems in radiology. Clear rules must be established on how the provider must inform the deployer. ESR recommends the use of AI model cards to increase transparency by providing detailed documentation on a model’s development, capabilities and limitations, ensuring that users are well-informed of operational characteristics and potential biases [13].

## Human oversight

Article 14 of the AI Act mandates human oversight of AI systems. In the context of diagnosis and treatment, adequate oversight of high-risk AI systems is paramount to ensure the patients’ trust in healthcare professionals using AI systems and delivery of a high standard of care. Regulations should define the professional role of the human overseeing each AI application. Generally, the overseeing person should be adequately trained for the task that the AI addresses (i.e., applications for image acquisition, processing, post-processing and interpretation may require radiologist oversight). However, some AI applications in healthcare may pose unrecognised risks, as research into human-AI interaction is still developing. While AI systems could prove useful in mitigating the foreseeable workforce shortages, it should be recognised that with ever-increasing workloads and cognitive efforts required from healthcare professionals, the risk of automation bias and overreliance may arise [14].

Similarly, such psychological effects could lead to deskilling effects in the workforce over time, which could prove detrimental to patients in situations where AI systems fail or become unavailable. These effects could be especially felt in radiology, where digitisation has led to a substantial increase in productivity over the past years and thus to higher mental loads per workday [15, 16]. In radiology, but also in healthcare more broadly, these phenomena should be carefully studied to aid in defining appropriate measures to ensure adequate human oversight and develop training programs that enable users to properly understand the AI systems being used and correctly interpret and contextualise their output.

In light of the complexities and challenges surrounding the integration of AI into healthcare, it is imperative to take proactive measures to ensure the effective utilisation of these technologies, identifying risks while safeguarding patient care. To this end, we recommend enhancing the training of radiologists by including comprehensive education on AI systems. Additionally, investing in research into mental load in healthcare settings can help determine thresholds for effective human oversight. Developing surrogate measures to evaluate oversight quality is also vital as well as implementing regular training and re-certification programs for radiologists, with or without AI, can prevent deskilling and ensure ongoing competency. These steps will empower healthcare professionals, improve patient care, and navigate the complexities of AI integration effectively.

### Recommendation

The ESR is aware of workforce issues in radiology and actively engages in projects regarding training levels of the radiological workforce (e.g., EU-REST). Similar efforts should be considered with special attention to the overall mental load in radiology as scientific evidence is needed to establish how effective human oversight can be exercised when dealing with high-risk medical AI solutions. Furthermore, the ESR would like to offer its support in establishing guidelines for necessary qualifications (see also AI literacy above) to effectively oversee AI in radiology and medicine, in general, depending on the specific use-case at hand (see also Classification Rules for High-Risk AI Systems).

## Quality management system

A quality management system (QMS) is a formalised framework that documents processes, procedures, and necessary responsibilities to achieve predefined quality policies and objectives. To market a medical device in the EU, manufacturers must obtain CE marking by adhering to the regulations outlined in the European Union Medical Device Regulation (EU MDR). The EU MDR specifies the requirements for a QMS, emphasising the safety, efficacy and efficiency of medical devices. It also mandates a post-market surveillance system and post-market clinical follow-up for each medical device.

The AI Act, Article 17, introduces QMS requirements that broadly align with requirements of the EU MDR, including data governance, algorithmic transparency, risk management, bias mitigation, ethical considerations on the use of AI, communication with relevant authorities and stakeholders, resource management, and clear delineation of responsibilities for staff and management.

### Recommendation

The requirement for distinct QMSs to comply with the EU MDR and EU AI Act may complicate the interpretation of the specific processes and requirements under each regulation. To address this, we call for greater harmonisation between the QMS requirements for both regulations—a notion that is also supported by COCIR, the European Trade Association representing medical imaging, radiotherapy, health ICT and electromedical industries [17]. A unified documentation process for vendors marketing AI tools as ‘high risk’ medical devices (according to EU AI Act classification) would streamline compliance, ensuring clarity and consistency. This approach would facilitate interpretation and ensure relevant and correct standards are met before the implementation of AI tools for medical imaging.

## Obligations of deployers of high-risk AI systems

Any radiologist or imaging department using AI in the clinical setting is considered a “deployer” in the context of the AI Act and, thereby, is subject to specific obligations, as defined by Article 26 of the AI Act. First, deployers must ensure that high-risk systems are only used according to the provider’s instructions and under human supervision, supervise the relevance and appropriateness of input data, and operate monitoring systems. In other words, the responsibility for AI applications remains in the hands of the caretakers. The AI Act gives this central ethical claim about responsibility in the clinical context of AI application a legal frame and determines the medical staff as key actors. Other ethical strategies about responsibility for automated applications, like public or industrial responsibility, are moved to the background. Second, deployers must inform patients and employees about any use of high-risk AI and store the results or log files. Furthermore, radiological deployers must inform the provider and the competent authorities immediately if a risk is identified. Operators must also conduct data protection checks and cooperate with the competent authorities. These administrative obligations imply steady surveillance and quality assurance measures which need further specification so that deployers across Europe exercise them in a harmonised manner, unless national law requires additional measures.

### Recommendation

The ESR offers to support deployers with its expertise, providing deployment recommendations and monitoring strategies in collaboration with providers. Furthermore, the ESR offers to be involved in any efforts to further specify deployers’ obligations in the field of radiology.

## AI regulatory sandboxes

Article 57 of the AI Act allows the development of regulatory sandboxes for the testing of innovative AI products that do not fall under current regulatory activities, fostering innovation while allowing AI implementation in a controlled environment. Despite the apparent benefits linked to controlled testing of innovative AI solutions without applying the current regulations, important considerations are raised with regard to AI systems related to radiology. Efficient testing of radiology-related algorithms requires data collection from several international sites, encompassing a wide range of radiological technologies and a diverse patient population.

Relying on regulatory sandboxes for large-scale operations can present significant challenges. In essence, when testing happens at a large scale, operating under limited regulation can imply risks related to patient safety (e.g., consequences of failed algorithm results) and privacy. Therefore, establishing regulatory sandboxes at a national level, as posed in paragraph 1 of Article 57 will not be enough for the efficient testing of medical/radiological AI. AI products should be viewed as health interventions and should be handled with the same regulations as any other intervention related to patient health. Large scale multi-centre clinical studies should be required to roll out any AI product that can affect patient health and should be treated as any other medical device regarding regulatory aspects. Transparency and rigorous evaluation of AI products are of utmost importance and should be prioritised over short testing rounds.

As proposed in Article 57, national competent authorities must have sufficient AI expertise to be able to oversee the function of sandboxes. The unique intricacies of radiological AI require oversight by medically-trained regulators with expertise in radiology and AI, to account for issues related to radiation protection and to assess the impact of AI algorithm failure on individual patient care and the healthcare system. These experts must define specific criteria and endpoints in the testing process, relevant to the unique risk of medical imaging AI.

As in other AI domains, access to potential regulatory sandboxes should be democratised allowing the participation of small and medium-sized enterprises (SMEs) as well as large companies in the field. The majority of AI companies in the field of radiological diagnostics fall into the SME category and should have equal access to AI regulatory sandboxes.

### Recommendation

For any clinical product, high-quality studies (i.e., peer-reviewed, multicentre, adequately powered, respecting data diversity) should be required prior to receiving regulatory approval. Image-based AI requires large datasets which defy the concept of “limited testing” within regulatory sandboxes but also high-quality studies. The composition of national competent authorities which oversee these sandboxes needs to be clearly defined based on the operation domain of each AI algorithm. In the case of imaging applications, experts in medical imaging AI should be members of the national authorities. Care needs to be taken so that SMEs have equal access to regulatory sandboxes compared to large enterprises.

## Post-market monitoring, information sharing, market surveillance

Article 72 of the AI Act mandates that providers establish a post-market monitoring system proportionate to the nature of the high-risk AI system. Importantly, data on the performance of high-risk AI systems should be collected either by the providers or the deployers of the AI system. This is especially important in cases where the AI system is used to support clinical reasoning and diagnostic or prognostic decision-making, where suggestions of the AI system—correct or incorrect—could influence the clinical decision-making of the human healthcare professional and consequently lead to health risks for patients [18, 19]. Furthermore, there is a need for clarification regarding the regulations for “adaptive” or “self-learning” systems, as these are currently not defined in Operational Obligations of Notified Bodies (Article 34). Structural deviation of performance metrics, whether on a group or subgroup level, due to model or data drift, might only accurately be captured by longitudinal collection and structured analysis of image-level or patient-level performance metrics, although standard practices have yet to be developed [20].

In addition, patient-level outcome metrics in radiology can be especially challenging to collect due to several factors. Depending on the use case, health risks may only manifest after longer time periods, such as missed lesions that turn out to be cancer. In some instances, these outcomes may seem independent of the AI system’s usage but are actually caused by changes in the patient’s management—like false-positive findings leading to additional diagnostic or treatment decisions that carry their own risks. Lastly, certain effects may only be discovered upon analysis of larger datasets, such as small percentage changes on specific events that might seem spurious in smaller-scale observations. To be meaningful and actionable, post-market surveillance strategies should be developed and adapted to the specific use-cases individually, and relevant metrics should be chosen together with domain experts [21, 22].

Furthermore, digital healthcare infrastructures must be designed to support population-level monitoring and fulfilment of AI companies’ duties. These processes are resource- and time-intensive, falling outside the available resources of departments. Consequently, the end-user must not be solely burdened with post-market monitoring responsibility.

### Recommendation

To enable effective post-market monitoring of AI systems, infrastructures need to be created that allow to track AI usage and potential detrimental effects to individual patients and/or larger patient populations. As outlined in the section on “Data and Data Governance” above, the ESR believes that in the long term the EHDS could serve as an infrastructure to facilitate collecting the relevant data. Therefore, the ESR would like to contribute its expertise to work towards interoperable and reliable technical solutions to facilitate post-market surveillance on a broader population level. Similarly, the ESR would like to offer its collaboration to efforts defining which data needs to be collected for which use-case. Lastly, the ESR acknowledges that European Commission project funding for this scope would significantly enhance the capacity to effectively address these challenges, also in alignment with the requirements of the EHDS.

## Conclusion and recommendations

The European Society of Radiology (ESR) remains steadfast in its commitment to advancing the safe and effective integration of AI into medical imaging. Our analysis aims to underscore several elements from different domains to ensure the ethical and beneficial deployment of AI technologies in radiology, enhancing diagnostic accuracy, patient care, and operational efficiency. Thus, we recommend several key actions and define our support with a special focus on the points listed below.

The ESR calls upon the European Commission to deliver implementation guidelines for the medical sector to support stakeholders with the implementation of the new regulatory requirements. These guidelines should be developed in partnership and consultation with relevant stakeholders. The ESR is prepared to support the European Commission in the implementation and development of AI guidelines specific to the medical imaging sector to ensure that AI technologies are integrated responsibly and effectively.

## Abbreviations

AI: Artificial intelligence
AI Act: Artificial Intelligence Act
CE: Conformité Européenne
EHDS: European Health Data Space
ESR: European Society of Radiology
COCIR: European Trade Association representing medical imaging, radiotherapy, health ICT, and electromedical industries
EU: European Union
EU MDR: European Union Medical Device Regulation
QMS: Quality management system
SME: Small and medium-sized enterprises
